# Ebola and COVID-19 in Democratic Republic of Congo: grappling with two plagues at once

**DOI:** 10.1186/s41182-021-00356-6

**Published:** 2021-08-24

**Authors:** Fatima Muhammad Asad Khan, Mohammad Mehedi Hasan, Zohra Kazmi, Ana Carla dos Santos Costa, Abdullahi Tunde Aborode, Shoaib Ahmad, Mohammad Yasir Essar

**Affiliations:** 1 Dow University of Health Sciences, Karachi, Pakistan; 2 Department of Biochemistry and Molecular Biology, Faculty of Life Science, Mawlana Bhashani Science and Technology University, Tangail, Bangladesh; 3 Division of Infectious Diseases, The Red-Green Research Centre, BICCB, Dhaka, Bangladesh; 4 Jinnah Medical and Dental College, Karachi, Pakistan; 5 Faculty of Medicine, Federal University of Bahia, Salvador, Bahia Brazil; 6 Healthy Africans Platform, Research and Development, Ibadan, Nigeria; 7 West African Academy of Public Health, Research and Development, Abuja, Nigeria; 8 Department of Surgery, District Head Quarters Teaching Hospital, Faisalabad, Pakistan; 9 Medical Research Center, Kateb University, Kabul, Afghanistan

**Keywords:** Ebola, COVID-19, Democratic Republic of Congo, Outbreak, Pandemic, Public health

## Abstract

In February 2021, a new Ebola virus disease outbreak was confirmed amid the COVID-19 pandemic in the Democratic Republic of Congo. Although the country has successfully contained the outbreak amid its fight against the COVID-19 pandemic, the epidemiological situation is still concerning, primarily due to the risk of an increase in the number of COVID-19 cases. The coexistence of both outbreaks increased the burden on the country’s health system mainly because Ebola response programs were redirected to the COVID-19 national response. Strategies adopted and lessons learned from previous Ebola outbreaks were crucial to developing the COVID-19 national response. To tackle the challenges of combating both the viruses, it is essential to adopt multidisciplinary measures such as prevention, education, and vaccination campaigns, promoting hygiene and social distancing practices, and improving diagnostic and management protocols. This paper discusses the efforts, challenges, and possible solutions to grapple with Ebola amid the COVID-19 crisis in DRC successfully.

To the editor,

Democratic Republic of Congo’s (DRC’s) largest-ever outbreak of Ebola virus disease (EVD) spanned from August 2018 to June 2020, which recorded 3481 cases and 2299 deaths in North Kivu, South Kivu, and Ituri provinces [1]. The current EVD outbreak is the DRC’s fourth occurrence in less than 3 years, which was announced on February 7, 2021, when a new Ebola case was confirmed in North Kivu province. Consequently, the DRC Ministry of Health took immediate action. The outbreak was declared over on May 3, 2021, by the World Health Organization (WHO), after resulting in six deaths and 12 cases in the country [2].

Although DRC has successfully contained the Ebola outbreak, the country’s epidemiological situation needs to be closely monitored. The data from May 15, 2021, suggest that the percentage of COVID-19 cases in DRC has dropped by almost 30% in the last week; however, WHO still reported 271 new cases and three deaths within that period [3]. In addition to COVID-19, the risk of other communicable diseases, such as cholera and measles, are further overwhelming the DRC health system, which may compromise the nation’s control efforts to quickly detect and respond to new EVD cases [4, 5]. This paper discusses the implications of the latest Ebola outbreak amid the COVID-19 pandemic in DRC, delineates the present challenges to public health security in the country, and provides subsequent suggestions to combat the dual challenge of two deadly pathogens.

It is noteworthy to mention that a hostile climate, poor road networks, ruthless terrain of deep rainforests and savannas, and extreme poverty make DRC’s public health infrastructure eminently vulnerable [6]. In the past, political instability, armed conflict, a deep mistrust towards local authorities, and the subsequent lack of community engagement have proven to be the main obstacles in reducing EVD outbreaks in the DRC. Additionally, cultural practices, such as unsafe burials and increased hesitancy to seek timely healthcare, have allowed Ebola to spread rampantly across communities [7].

The COVID-19 pandemic negatively impacted the epidemiological control of several infectious diseases worldwide [8–10]. For instance, Africa is facing a spread of Yellow fever, Lassa fever, measles, and arboviruses amidst the pandemic [5, 11–13]. Even before the 2021’s outbreak, reports anticipated an accompanying surge in Ebola occurrences amidst COVID-19 in Africa, as numerous Ebola community outreach strategies were ceased [14]. The imposition of strict lockdowns threatened Ebola control activities, leading to inefficient Ebola surveillance and contact tracing [15, 16]. In addition, the national leadership of the Ebola response was also made responsible for supervising DRC’s COVID-19 response; hence, various Ebola response workforces were redirected to containing the COVID-19 crisis. This decline in workforces, measures and attention placed the continuity of efficient EVD surveillance in considerable danger [17].

Although COVID-19 has introduced significant obstacles that threaten the health, wealth, and social structure of DRC, several lessons learned from managing the previous calamitous Ebola virus eruptions have been crucial in initiating the existing COVID-19 health responses. These include collaborating with community representatives and organizations to elucidate the disease and circulate important information to the public in native languages. The foundations and workforces of the Ebola virus have been redistributed to the COVID-19 response. UNICEF redirects additional staff, provides medical tools for Ebola cases in North Kivu, and works closely with local health organizations to contribute to source control and disinfecting activities [18].

Vaccines are essential to strengthen immunity against both COVID-19 and Ebola virus disease. More than 1.7 million vaccines of COVID-19 have been delivered to the capital city of Kinshasa by COVAX. AstraZeneca vaccine is being used because it meets the storage circumstances, i.e., 2–8 °C in DRC [19]. The country has also inaugurated the Butembo Vaccination drive against Ebola. Almost 8000 doses of the Ervebo vaccine, the primary vaccine to be endorsed by the US Food and Drug Administration in December 2019, are being used [20].

The failures and challenges encountered in dealing with the recent Ebola outbreak in the DRC point towards the need for more comprehensive measures to guarantee a more effective national response, both in the prevention and control of future outbreaks of Ebola and other infectious diseases in the country. The first point to be addressed is the need for a greater understanding of the gaps and flaws of health security in the DRC. For this, it is necessary to reassess the congruence between the measures of prevention, detection, and response to Ebola outbreaks and other acute public health emergencies adopted by the country and the recommendations of the International Health Regulations (IHR) of the World Health Organization. It is necessary that the monitoring and evaluation of the recommendations of the IHR through the voluntary Joint External Evaluation (JEE) is carried out continuously and frequently, following the outbreaks of re-emerging infections, and especially in public health crises that provide a favorable environment for the occurrence of new emergencies, such as the COVID-19 pandemic, humanitarian disasters, and conflicts. This is essential to determine the actual capacity of the DRC, in the present context, to prepare and respond to future public health threats through a coordinated response.

The JEE is also considered necessary to guide the effective redirection of monetary, human and infrastructure resources, preventing public health emergencies from becoming environments conducive to the resurgence of previously controlled diseases due to the uncoordinated reallocation and prioritization of resources. The responsible institutional bodies must establish well-defined health safety protocols regarding the necessary actions to prioritize resources and address any gaps.

The second point is the planning strategy, which must focus on preventing, detecting, and responding to the risks of new Ebola outbreaks. For prevention, it is essential to strengthen warning systems to detect the occurrence of isolated cases of Ebola as soon as possible and begin contact tracing to prevent the spread of the disease, such as isolation of suspected individuals and those who have had contact with someone with a suspected or proven diagnosis of Ebola.

Furthermore, a strengthened health care strategy is necessary for DRC since conflicts, political turmoil, and current lockdowns and social distancing protocols due to COVID-19 have made it extremely difficult to access health services in some areas and communities. Active and passive surveillance within each community should be carried out, especially those that pose a greater risk for the resurgence of Ebola cases. Telemedicine services also play an essential role in surveillance, either by monitoring suspected individuals in locations with difficult access or allowing collaboration between specialists trained in identifying and managing the disease and health professionals with a comparatively lower level of expertise.

Conducting robust epidemiological analysis based on previous outbreaks is critical in anticipating new cases and preventing future outbreaks. Since genetic analysis confirmed that the index case of the last EVD outbreak was related to an EVD survivor from the 2018–2020 epidemic [21], and EVD relapses have also been reported in vaccinated individuals [22], additional research to assess the scientific origins of this outbreak should be carried out. In addition, the findings cited points to keep track of recovered patients, and individuals vaccinated against Ebola, identify probable relapses of the disease, and recognize risk factors that may be involved in this process.

As past outbreaks have demonstrated the critical importance of social mobilization to reduce EVD transmission in the community, it is also necessary to improve communication with the population and the dissemination of correct information to demystify false news and fight disease-related stigma, which can lead to greater hesitation in seeking timely medical care. It is also desirable to enable risk communication in local languages for rapid identification of warning symptoms. This can be done by seeking support from local leaders, particularly religious leaders and other important groups in the community.

Such health education campaigns should also emphasize prevention strategies and clinical characteristics of Ebola virus transmission, reaching as many people as possible, especially populations at risk. The media and social networks can also disseminate information and guide people on the measures to be taken in case of clinical suspicion or contact with the virus. It is necessary to reinforce the importance of notifying cases and isolating possible suspicions, as well as the need for strict hygiene and safety protocols to be used while burying the victims of the disease.

Since adopting a preventive approach is cost-effective in places of difficult epidemiological control during outbreaks, developing an effective and accessible vaccine against EVD would be the central pillar of prevention against the disease, especially in contexts of health challenges, such as the COVID-19 pandemic. Highly effective vaccines are now available for Ebola virus disease and were instrumental in ending this epidemic in just 3 months. Therefore, there is a need to implement EVD vaccination strategies in at-risk communities. As a country at high risk for Ebola outbreaks, to ensure a rapid response in cases of imminent disease, national and international coordination groups must maintain a stock of EVD vaccines and distribution protocols and conduct emergency vaccination campaigns in accessible and convenient centers, especially in areas susceptible to outbreaks.

The confluence of two lethal viruses, COVID-19 and Ebola, serves as a potent reminder of the necessity to reinforce medical and public health capacities in DRC. While the latest EVD outbreak is over, the threat of future outbreaks should not be underestimated. As the nation strives to recover from the social and economic toll of the pandemic, it is important that the leadership keeps financing the health system in a dedicated effort to improve surveillance, testing and tracing capacities, awareness programs and protective vaccination campaigns. It is essential to adopt multidisciplinary measures such as promotion of hygiene and social distancing practices, and improvement of diagnostic and management protocols, to coordinate a timely response to future EVD outbreaks while ensuring an equitable distribution of resources between the country’s two crises.

## Data Availability

Not applicable.

## Abbreviations

DRC: Democratic Republic of Congo
EVD: Ebola virus disease
WHO: World Health Organization
COVID-19: Coronavirus disease 2019
IHR: International Health Regulations
JEE: Joint External Evaluation

## References

[1] World Health Organization. Ebola virus disease. 2021. https://www.who.int/en/news-room/fact-sheets/detail/ebola-virus-disease. Accessed 18 May 2021.

[2] Centers for Disease Control and Prevention. Ebola (Ebola Virus Disease)|2021 Democratic Republic of the Congo, North Kivu Province. 2021. https://www.cdc.gov/vhf/ebola/outbreaks/drc/2021-february.html. Accessed 18 May 2021.

[3] World Health Organization. Democratic Republic of the Congo: WHO coronavirus disease (COVID-19) dashboard with vaccination data. 2021. https://covid19.who.int/region/afro/country/cd. Accessed 18 May 2021.

[4] World Health Organization. Ebola virus disease—Democratic Republic of the Congo. 2021. https://www.who.int/csr/don/10-february-2021-ebola-drc/en/. Accessed 18 May 2021.

[5] Mohan A, Temitope RA, Çavdaroğlu S, Hasan MM, Costa ACdS, Ahmad S, Essar MY (2021). Measles returns to the Democratic Republic of Congo: a new predicament amid the COVID-19 crisis. J Med Virol.

[6] Nzolo D, Kuemmerle A, Lula Y, Ntamabyaliro N, Engo A, Mvete B, Liwono J, Lusakibanza M, Mesia G, Burri C, Mampunza S, Tona G (2019). Development of a pharmacovigilance system in a resource-limited country: the experience of the Democratic Republic of Congo. Ther Adv Drug Saf.

[7] Aruna A, Mbala P, Minikulu L, Mukadi D, Bulemfu D, Edidi F, Bulabula J, Tshapenda G, Nsio J, Kitenge R, Mbuyi G, Mwanzembe C, Kombe J, Lubula L, Shako JC, Mossoko M, Mulangu F, Mutombo A, Sana E, Tutu Y, Kabange L, Makengo J, Tshibinkufua F, Ahuka-Mundeke S, Muyembe J-J, Alarcon W, Bonwitt J, Bugli D, Bustamante ND, Choi M, Dahl BA, DeCock K, Dismer A, Doshi R, Dubray C, Fitter D, Ghiselli M, Hall N, BenHamida A, McCollum AM, Neatherlin J, Raghunathan PL, Ravat F, Reynolds MG, Rico A, Smith N, Soke GN, Trudeau AT, Victory KR, Worrell MC, ER CDC (2019). Ebola virus disease outbreak Democratic Republic of the Congo, August 2018–November 2019. MMWR Morb Mortal Wkly Rep.

[8] Costa ACdS, Hasan MM, Xenophontos E, Mohanan P, Edet BE, Hashim HT, Ahmad S, Essar MY (2021). COVID-19 and zika: an emerging dilemma for Brazil. J Med Virol.

[9] Yousaf A, Khan FMA, Hasan MM, Ullah I, Bardhan M (2021). Dengue, measles, and COVID-19: a threefold challenge to public health security in Pakistan, Ethics. Med Public Health.

[10] Rocha ICN, Hasan MM, Goyal S, Patel T, Jain S, Ghosh A, Cedeño TDD (2021). COVID-19 and mucormycosis syndemic: double health threat to a collapsing healthcare system in India. Trop Med Int Health.

[11] Hasan MM, Costa ACdS, Xenophontos E, Mohanan P, Bassey EE, Ahmad S, Essar MY (2021). Lassa fever and COVID-19 in Africa: a double crisis on the fragile health system. J Med Virol.

[12] Uwishema O, Adanur I, Babatunde AO, Hasan MM, Elmahi OKO, Olajumoke KB, Aborode AT, Emmanuella N, Costa ACdS, Ahmad S, Essar MY (2021). Viral infections amidst COVID-19 in Africa: implications and recommendations. J Med Virol.

[13] Çavdaroğlu S, Hasan MM, Mohan A, Xenophontos E, Costa ACdS, Aborode AT, Tsagkaris C, Outani O, Ahmad S, Essar MY (2021). The spread of Yellow fever amidst the COVID-19 pandemic in Africa and the ongoing efforts to mitigate it. J Med Virol.

[14] Aborode AT, Tsagkaris C, Jain S, Ahmad S, Essar MY, Fajemisin EA, Adanur I, Uwishema O (2021). Ebola outbreak amid COVID-19 in the Republic of Guinea: priorities for achieving control. Am J Trop Med Hyg.

[15] Devex. The double burden of coronavirus and Ebola in eastern DRC. 2021. https://www.devex.com/news/the-double-burden-of-coronavirus-and-ebola-in-eastern-drc-97189. Accessed 18 May 2021.

[16] Médecins Sans Frontières. DRC tenth Ebola outbreak. 2021. https://www.msf.org/drc-tenth-ebola-outbreak. Accessed 18 May 2021.

[17] Christie A, Neatherlin JC, Nichol ST, Beach M, Redfield RR (2020). Ebola response priorities in the time of Covid-19. N Engl J Med.

[18] UNICEF. UNICEF deploys staff, medical equipment and supplies in response to new Ebola case in eastern DRC. 2021. https://www.unicef.org/press-releases/unicef-deploys-staff-medical-equipment-and-supplies-response-new-ebola-case-eastern. Accessed 19 May 2021.

[19] UNICEF. More than 1.7 million COVID-19 vaccines arrive in the Democratic Republic of Congo. 2021. https://www.unicef.org/press-releases/more-17-million-covid-19-vaccines-arrive-democratic-republic-congo. Accessed 19 May 2021.

[20] BBC News. Ebola: DR Congo launches Butembo vaccination campaign. 2021. https://www.bbc.com/news/world-africa-56073201. Accessed 19 May 2021.

[21] World Health Organization. Resurgence of Ebola in North Kivu in the Democratic Republic of the Congo. 2021. https://www.afro.who.int/news/resurgence-ebola-north-kivu-democratic-republic-congo. Accessed 25 July 2021.

[22] Mbala-Kingebeni P, Pratt C, Mutafali-Ruffin M, Pauthner MG, Bile F, Nkuba-Ndaye A, Black A, Kinganda-Lusamaki E, Faye M, Aziza A, Diagne MM, Mukadi D, White B, Hadfield J, Gangavarapu K, Bisento N, Kazadi D, Nsunda B, Akonga M, Tshiani O, Misasi J, Ploquin A, Epaso V, Sana-Paka E, N’kasar YTT, Mambu F, Edidi F, Matondo M, Bula JB, Diallo B, Keita M, Belizaire MRD, Fall IS, Yam A, Mulangu S, Rimion AW, Salfati E, Torkamani A, Suchard MA, Crozier I, Hensley L, Rambaut A, Faye O, Sall A, Sullivan NJ, Bedford T, Andersen KG, Wiley MR, Ahuka-Mundeke S, Tamfum J-JM (2021). Ebola virus transmission initiated by relapse of systemic ebola virus disease. N Engl J Med.

